# 916. Finding the Missing Millions and Addressing Health Disparities: Automated Hepatitis B Screening and Linkage to Care

**DOI:** 10.1093/ofid/ofab466.1111

**Published:** 2021-12-04

**Authors:** Binghong Xu, Su Wang, Ruth P Brogden, Jaymie Yango, Mary O Adedeji

**Affiliations:** 1 Center for Asian Health, RWJBH-Saint Barnabas Medical Center, Florham Park, New Jersey; 2 Saint Barnabas Medical Center & World Hepatitis Alliance, New Providence, New Jersey; 3 RWJBH-Saint Barnabas Medical Center, Florham Park, New Jersey; 4 RWJBH - Saint Barnabas Medical Center, Livingston, New Jersey; 5 Saint Barnabas Medical Center, Hillside, New Jersey

## Abstract

**Background:**

Globally, HBV is the most common blood-borne infection. An estimated 1.2 million people in the US and 350 million worldwide lived with HBV, a primary driver of liver cancer. It is endemic in many parts of the world and is a major health disparity in immigrant communities, including the US, which has the largest immigrant population in the world. Asian American Pacific Islanders are 5% of the total population in the US, but represent 50% of people living with HBV. In 2016, WHO set a goal of hepatitis elimination by 2030 but with only 10% of those living with HBV diagnosed, screening must be scaled up.

**Methods:**

Modifications were made in the electronic medical record (EMR) to automate screening, with HBV (HBsAg) orders triggered by a patient’s country of birth or race. The began in the Emergency Department and later expanded to the Inpatient setting. Automated notifications are sent to nurse for eligible patients and then to the patient navigator (PN) for positive tests. The PN contacts the patient to provide education and arrange linkage-to-care (LTC) for evaluation and care.

**Results:**

From Mar 2018 to Mar 2021, we conducted 23,883 HBV screenings. The patients originated from 173 countries based on registration; top 5 countries of origin were Haiti, Jamaica, Ecuador, Guyana, and Portugal. We found 228 (1.0%) patients with HBV infection, 101 (47%) were newly diagnosed and 182 (85%) were linked to care. We examined race and insurance status for any association with those previously tested versus newly diagnosed. Blacks were more likely to be newly identified HBV versus Asians (61.6% vs. 28.9%, p< .001), as were self-pay (uninsured) versus insured patients (66.7% vs 47.2%, p=0.043). Compared to the approximately 0.4% HBV prevalence in the US, the HBV prevalence in several towns around our hospital in Essex County is two to four times higher.

Table 1. The HBV Prevalence in Towns of Essex County

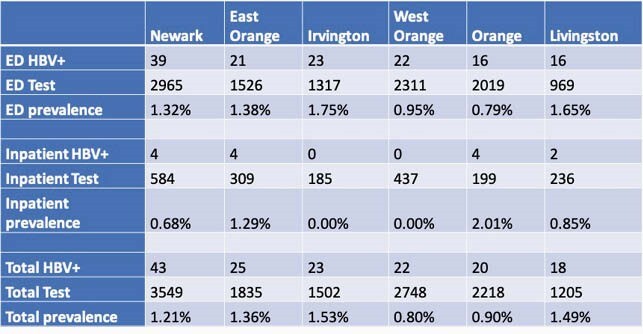

**Conclusion:**

Our community is diverse and social determinants of health, like race and insurance status, may contribute to provider behaviors of HBV screening with blacks receiving less screening than Asians. Automated testing programs can address health disparities and scale up screening. Such micro-elimination approaches are important for achieving global hepatitis elimination by 2030.

**Disclosures:**

**Su Wang, MD MPH**, **Gilead Sciences** (Grant/Research Support)**Gilead Sciences** (Grant/Research Support)

